# Vaginal Caesarian Section

**Published:** 1910-11

**Authors:** R. R. Kime

**Affiliations:** Atlanta, Ga.


### VAGINAL CAESAREAN SECTION.

### *Indications, Limitations and Technique.*

By R. R. Kime., M. D., Atlanta, Ga.

Vaginal Caesarean Section is of recent origin, being first performed by Duhrssen in 1895 and lately popularized as a recognized obstetric procedure in certain cases. In order to more definitely ascertain its limitations, we will first briefly consider the general indications for Caesarean section and its various modifications to meet individual cases.

Complications of pregnancy indicating Caesarean section can be briefly summarized as follows:—

a—Deformity of pelvis.

b—Tumors of lower segment of uterus, fixed in pelvis.

c—Eclampsid.

d—Placenta Praevia.

e—Cancer cervix.

f—Rigid, misplaced or cicatrical cervix in cases demanding rapid delivery.

To meet these indications, we have Caesarean section in some of its modified forms such as—

1—Classical Abdominal Caesarean Section.

2—Poro-operation.

3—Caesarean Section, Complete Hysterectomy, Hysterotomy and Pan-Hysterectomy.

4—Supra-public extra-peritoneal hysterotomy.

5—Vaginal Caesarean Section or Hysterotomy.

Sterilization of the mother may follow the second and third, while it may be elected in the first and fourth if the circumstances justify such a procedure, in the fifth it is never justifiable.

If complication (a) i. e., deformity of the pelvis precludes delivery of a normal living child, then the classical abdominal operation is indicated, if infection be present, then modification 2-3 or 4 is indicated. In complication (b) if the tumor is small and low in pelvis and easily reached, vaginal hysterotomy is indicated, if large and high in pelvis, then abdominal hysterotomy with removal of tumor is indicated and if infection be present, hysterectomy.

If tumor is not fixed in pelvis, nature will frequently correct the trouble and allow the child to pass, especially if favored by the position of the patient and proper manipulation by the accoucher.

In Eclampsia (c) we have more positive indications for vaginal hysterotomy.

At the present time most writers agree that Eclampsia is due to a toxaemia, and if allowed to continue will produce serious changes not only in the kidneys but also in the liver, heart, brain, etc., and will seriously affect the foetus in utero.

It is also conceded that the active treatment of Eclampsia is 50 per cent less fatal than the expectant treatment. In round numbers the death rate is reported in expectant treatment as 20 to 30 per cent, and in active treatment as 10 to 15 per cent.

Vaginal Hysterotomy in the hands of some has very materially reduced this rate.

Fry—(Amer. Jour. Obs.) reports fifteen cases with one death.

Veit 33 cases with one death. (Peterson).

Bumm—37 cases, deaths not given. (Peterson).

The active treatment of Puerperal Eclampsia depends upon the period of pregnancy, stage of labor, size of pelvis, condition of soft parts, and severity of attack. In Multipara, labor in progress, or easy dilatable, no especial obstruction to delivery hysterotomy is not indicated. If cervix is not dilatable, the attack mild, regaining consciousness quickly, even then hysterotomy is not justifiable. The convulsions can be controlled with veratrium, apomorphia or hyosine, and Morph., temporarily avoid continued use of chloroform while elimination is set up, bowels moved, membranes ruptured, and lower segment of uterus and vagina packed well with steirle gauze wet in sterile boric acid solution. Carry plenty of gauze (3 or 4 in. wide strips best), high up in uterus and plenty of it. Then throw gr. x x quinine sulph., in rectum or give gr v every hour by mouth to stimulate uterine contractions, and many cases will be delivered naturally in a few hours safely without doing patient harm. In cases of profound toxaemia, cervix rigid, then vaginal hysterotomy is the operation of choice unless some pelvic obstruction exists or pregnancy has gone over time with a large head and excessive ossification of skull bones, then abdominal hysterotomy is the operation of election in order to save both mother and child.

The forceable instrumental dilation of the cervix in these cases by the Bossi pattern is rarely if ever justifiable because of bruising and tearing of tissues. Any case that can not be safely handled by dilatation with the fingers or by use of the gauze tampon as described in a case for vaginal hysterotomy.

In case of uterine infection complicating Eclampsia the same rules apply as above, remembering that there is less shock and that the peritoneal cavity is not opened in vaginal hysterotomy.

If pregnancy is terminated before the full term, then the indication is more positive for the vaginal operation.

In cases of Placenta Praevia (d) if the cervix will admit the hand, immediate podalic version is the operation of choice.

If cervix is small the parts should be disinfected and the sterile antiseptic gauze tampon used with a vulval dressing and a firm bandage applied so that tampon will not be forced out by vomiting or coughing. In the meantime the patient can be prepared for operation. If after 18 to 24 hrs., the cervix will not then admit the hand, then hysterotomy is indicated. If the patient is at or near full term, in the interest of both mother and child the abdominal operation as a rule should be done, if not at full term and placenta mostly on posterior wall or foetus dead, then vaginal hysterotomy is justifiable.

For the Cancer of Cervix (e) vaginal hysterotomy followed by vaginal hysterectomy is the operation of choice if such an operation is feasible.

The last indication (f) has been partially discussed, yet it is possible to have diseased or abnormal cervix that will not yield to the efforts of nature or has cicatricial deposits from previous operations, or so misplaced posteriorly from previous operations on body of uterus as to preclude normal delivery then in some instances vaginal hysterotomy will meet the indications best.

Vaginal Caesarean Section or Hysterotomy was evolved from vaginal operations on the uterine body in the field of gynecological work.

While I advise vaginal hysterotomy in certain conditions, it is advised on the same basis as abdominal hysterotomy and as a surgical operation of some magnitude designed to meet certain conditions and indications best.

Its field of usefulness is limited to where rapid delivery is demanded, the cervix not being easily dilated and there being no obstruction in pelvis or marked disproportion in size of child and pelvis that would preclude normal delivery.

I can not agree with some eminent obstetricians and gynecologists that every country practitioner should be prepared to do vaginal Caesarean section no more than he should do the abdominal operation which is certainly more frequently demanded.

Patient is prepared and put in same position as for vaginal hysterectomy in so far as time and circumstances will permit. Anterior lip of cervix is seized in each side near median line with heavy double tenaculae, cervix pulled well down—circular incision made on anterior half of cervix well through mucous membrane and below bladder wall-flap dissected up principally with finger and gauze blunt dissection exposing anterior wall of uterus well up to peritoneum, being careful not to injure peritoneum or bladder.

An anterior longitudinal incision of mucous membrane of vaginal wall will also be needed to expose parts well and prevent tearing of same in delivery. Incise anterior wall of cervix and uterus in median line with scissors up to height of dissection. If this does not give room enought for safe delivery, prepare and incise posterior wall in same way, thus increasing space for delivery without tearing or mutilating uterine tissues. Avoid entering abdominal cavity anteriorly or posteriorly if possible. If such an accident should occur, no serious harm is likely to occur if parts are properly sutured unless septic infection be present.

Child is delivered, preference being in favor of podalic version. Be certain to remove tenaculae from cervix before delivery to prevent injury to foetus or to maternal structures. Placenta should be removed promptly by Crede method. If much hemorrhage intra-uterine gauze tampon may be used but watch that it does not tend to produce convulsions after delivery by its presence. Close incision in uterus with No. 2 plain cat-gut by super imposed layers, continuous or interrupted sutures, avoid including of uterine mucosa. Close incisions of mucous membrane of vagina with interrupted suture so as to facilitate drainage and cover its raw surfaces. The incisions in the uterus and vagina represent an inverted cross also the sutures when completed. Williams makes the longitudinal incision in the anterior wall of the vagina, leaving off the transverse incision, the former vs. transverse and longitudinal combined I think gives most space. Pulling down well on cervix and incising in median line tends to lessen hemorrhage. A long, broad retractor to elevate bladder wall materially facilitates the dissection and incision of uterine wall as well as in the proper suturing of the tissues after delivery.

If the patient is a primipara, the vagina or perineum and vulva must be dilated before making incisions. If it is a full term pregnancy and large child, the posterior incision will be necessary.

In conclusion, I shall report one case typical in its indications for vaginal hysterotomy.

Patient—Colored, age 18—single—previous health good—stout hearty female. Taken early A. M. with convulsions recurring every 15 to 30 min., unconscious, unable to swallow, remaining unconscious all the time, some swelling of extremities. I saw patient about 9:30 A. M., in consultation.

Veratrium hyp.—Hyoscine 1-100 gr. Morph. 1/8 gr.—had very little effect on convulsions.

No labor pains, cervix rigid, small not dilatable, and rather high in pelvis. Kidneys acting very little—patient could not swallow cathartic. Advised vaginal Caesarean section and after some persuasion, patient was moved to Spellman Hospital and vaginal Caesarean section was performed as desribed in this paper. Patient was supposed to be 7½ or 8 months pregnant. Forceps were applied to head of foetus and required considerable force to deliver. Child could not be resuscitated and seemed to be full term. No convulsions after delivery, first specimen of urine coagulated two-thirds by bulk with heat and nitric acid, abundance of gran. and hyal. casts lucocytes and round epithelium. Temperature did not exceed 101 degrees F· after operation, normal on fourth day. No complications followed lying-in period, normal in every respect. Urine soon cleared up. Patient has been confined since and had a normal easy labor.

References.

Peterson, R.—Surg. Gyne. and Obs.—Vol. xi—Aug. 1910-210.
Peterson, R.—Surg. Gyne. and Obs.—Vol. viii—Feb. 1910-180
Humpstone, O. P.—Amer. Jour. Obs.—Vol. lix—Jan. 1909-92.
Fry, H. D.—Amer. Jour. Obs.—Vol. lix—Feb. 1909-202.
Porter, M. F.—Amer. Jour. Obs.—Vol. lxi—Jan. 1910-71..
Lewis, H. F.—Amer. Jour. Obs.—Vol. lx—Oct. 1909-586.
Sprig, M. W.—Amer. Jour. Obs.—Vol. lx—Oct· 1909-586.
Hirst, B. C.—Obstetrics—6th. Ed.—P. 795.
Williams, J. W.—Obstetrics—2nd. Ed.—P. 387.

